# Plant Growth Stimulators Improve Two Wheat Cultivars Salt-Tolerance: Insights into Their Physiological and Nutritional Responses

**DOI:** 10.3390/plants11233198

**Published:** 2022-11-22

**Authors:** Neveen B. Talaat, Alaa M. A. Hanafy

**Affiliations:** Department of Plant Physiology, Faculty of Agriculture, Cairo University, Giza 12613, Egypt

**Keywords:** photosynthetic pigments, ionic balance, osmotic adjustment, salicylic acid, salt tolerance, spermine, wheat (*Triticum aestivum* L.)

## Abstract

Spermine (SPM) and salicylic acid (SA), plant growth stimulators, are involved in various biological processes and responses to environmental cues in plants. However, the function of their combined treatment on wheat salt tolerance is unclear. In this study, wheat (*Triticum aestivum* L. cvs. Shandawel 1 and Sids 14) plants were grown under non-saline and saline (6.0 and 12.0 dS m^–1^) conditions and were foliar sprayed with 100 mgL^−1^ SA and/or 30 mgL^−1^ SPM. Exogenously applied SA and/or SPM relieved the adverse effects caused by salt stress and significantly improved wheat growth and production by inducing higher photosynthetic pigment (chlorophyll *a*, chlorophyll *b*, carotenoids) content, nutrient (N, P, K^+^, Ca^2+^, Mg^2+^, Fe, Zn, Cu) acquisition, ionic (K^+^/Na^+^, Ca^2+^/Na^+^, Mg^2+^/Na^+^) homeostatics, osmolyte (soluble sugars, free amino acids, proline, glycinebetaine) accumulation, protein content, along with significantly lower Na^+^ accumulation and chlorophyll *a*/*b* ratio. The best response was registered with SA and SPM combined treatment, especially in Shandawel 1. This study highlighted the recovery impact of SA and SPM combined treatment on salinity-damaged wheat plants. The newly discovered data demonstrate that this treatment significantly improved the photosynthetic pigment content, mineral homeostasis, and osmoprotector solutes buildup in salinity-damaged wheat plants. Therefore, it can be a better strategy for ameliorating salt toxicity in sustainable agricultural systems.

## 1. Introduction

Soil salinization is one of the most important environmental hazards that inhibits plant growth and development, causing significant yield losses. Approximately 50% of irrigated lands are suffered from the deleterious impact of salt stress [1]. Salinity affects plant growth through osmotic effect, nutritional imbalance, ionic toxicity, and oxidative stress [2]. The absorption of high concentrations of Na^+^ in cells limits K^+^ uptake, resulting in Na^+^ toxicity and nutrient imbalance during salt stress [1]. In order to establish the defense, plants restrict the accumulation of salt ions in the cytosol and enhance the synthesize of osmolytes [3,4,5]. Osmolyte biosynthesis is critical for maintaining osmotic potential, metabolic activity, and water uptake under saline conditions [6]. Additionally, enzymatic and non-enzymatic antioxidants can scavenge effectively the excessive reactive oxygen species (ROS) generated during salt stress [7,8,9,10,11]. Ionic status inside the plant cell is also very important for plant salt tolerance because the excess of salt ions in the cytoplasm disrupts ion homeostasis, inhibits plant growth, and affects water transport [7,12]. Excessive soil Na^+^ transported to the above ground organs disturbs intracellular ionic homeostasis in plants, damages photosynthetic membrane structure, promotes chlorophyll degradation, reduces photosynthetic efficiency, affects cytosolic enzyme activity, and inhibits cellular metabolism, all of which result in restrained plant growth and development [7,9,11]. The ability of plants to maintain a high cytosolic K^+^/Na^+^ ratio under ion toxicity is another salt tolerance mechanism [3,7].

Many plant growth regulators have been used to improve the salt tolerance of crops such as salicylic acid (SA). SA is a natural phenolic compound regulating plant growth and development under both normal and stressful conditions [13,14]. It has been reported that pretreatment with exogenous SA under saline environments prevents the chlorophyll degradation and increases the photosynthetic efficiency [13,15], reduces the ion toxicity, maintains the osmotic potential [7,13,16], stimulates the antioxidant enzymes, and scavenges the ROS in plants [10,15], as well as regulates the cellular signaling, senescence, and overall cellular redox homeostasis [15,16]. Moreover, an exogenous application of SA prevents lowering of indole acetic acid and cytokinin levels in salt-stressed wheat plants, thereby maintaining cell division and elongation processes in apical meristem of the roots resulting in an increase in growth and productivity of plants [17]. Previous report provides evidence that SA could alleviate salt stress through accelerating plant growth and improving leaf physiological processes [18]. In addition, exogenous SA balanced the osmotic potential and lowered the osmotic damage to the plasma membrane by mediating the accumulation profile of ions, such as Na^+^, K^+^, and Ca^2+^, as well as compatible metabolites, such as proline and soluble sugar [19]. Exogenous application of SA to wheat plants exposed to salt stress alleviated the deleterious effects of salinity by improving the photosynthetic pigment content and inducing the accumulation of certain osmolytes, such as soluble sugars, proline, and glycinebetaine [13]. A similar trend has been found in baby corn under salt stress in response to SA seed priming, which has been attributed to an increased ion content and an enhanced accumulation of certain osmolytes, such as proline [20]. Evidence also shows a promising role of SA foliar application against soil salinization in wheat plants through its effects on the content of polyamines [14]. Although there have been a lot of investigations study the effect of SA under salt stress, the physiological and biochemical mechanisms of SA in regulating wheat response to saline environments are still not fully understood.

Another class of biomolecules involved in plant stress response is composed of polyamines (PAs), which are low molecular weight aliphatic cations that are ubiquitous cellular components [21]. In plants, the major PAs—putrescine, spermidine, and spermine—have been shown to be involved in many aspects of plant growth and development, such as organogenesis, embryogenesis, flower initiation and development, leaf senescence, fruit development and ripening, as well as abiotic and biotic plant stress responses [21,22,23]. They can also act as anti-senescence and anti-stress agents due to their acid neutralizing and antioxidant properties, as well as for their membrane stabilizing abilities [24]. Among PAs, emphasis has been placed on the unique role of spermine (SPM, tetraamine) in the regulation of various defensive processes in plants. It has been described as a ‘potent plant defense activator with broad-spectrum protective effects’ when used exogenously [25]. The protective role of SPM in plants under salt stress has been reported. For example, the exogenously applied SPM alleviated the adverse effect of saline environments on plant development via maintaining the osmotic adjustment, protecting the structure and function of photosynthetic apparatus, maintaining the cationic-anionic stability, reducing the ethylene production, enhancing the protein content, modulating the endogenous phytohormone level, and inducing the organic solutes accumulation [23,26,27]. Additionally, previous study provides evidence that the SPM application could maintain significantly higher root vitality, leaf relative water content, photosynthesis, water use efficiency, osmolytes accumulation, K^+^/Na^+^ ratio, and antioxidant enzyme activity, as well as lower osmotic potential, Na^+^ accumulation, and oxidative damage in salt-stressed creeping bentgrass plants [28]. Recently, it has been shown that exogenous SPM treatment alleviated the inhibition of maize plant growth and productivity under stressful conditions by improving the antioxidant enzyme activity, antioxidant molecules content, and cell membrane integrity [29]. Although several researchers have studied the effects of exogenous SPM on enhancing plant abiotic stress tolerance, not much is known about the mechanism of SPM associated with wheat salt tolerance.

Among various food crops, wheat (*Triticum aestivum* L.) is a staple food in 30% of the world. However, it faces severe losses in its productivity due to salt stress [30,31]. Development of salt-tolerant wheat cultivars adapted to saline conditions is the most effective and economic strategy to combat this detrimental phenomenon. Exogenous treatment with plant defense elicitors, such as SA and SPM, is the best way to enhance plant salt tolerance. Some studies have looked into the effect of a single application of SA or SPM on plants exposed to salt stress. However, no one has looked into the impact of their combined treatment on wheat salt tolerance. To fill this gap, as a first investigation, we carried out this study to investigate the ameliorative impact of SA and SPM dual application to two wheat cultivars (*Triticum aestivum* L. cvs. Shandawel 1 and Sids 14) grown in salty soils through measuring the growth, productivity, photosynthetic pigment (chlorophyll *a*, chlorophyll *b*, carotenoids, total pigments) content, chlorophyll *a*/*b* ratio, nutrient (N, P, K^+^, Na^+^, Ca^2+^, Mg^2+^, Fe, Zn, Cu) acquisition, ionic (K^+^/Na^+^, Ca^2+^/Na^+^, Mg^2+^/Na^+^) homeostatic, osmoprotector solutes (total soluble sugars, total free amino acids, proline, glycinebetaine) accumulation, as well as grain carbohydrate and protein content. Accordingly, it has been hypothesized that the foliar application of SA and SPM combined treatment can help wheat plants to overcome the deleterious effects of salt stress on plant growth and production by enhancing photosynthetic pigment content, maintaining ionic homeostasis, adjusting osmotic balance, and increasing grain carbohydrate and protein content. This research provides novel insights into the mechanisms of SA- and SPM-mediated amelioration of salt stress on wheat.

## 2. Results

### 2.1. Foliar Applications of SA and/or SPM Ameliorate the Growth and Yield Reduction Caused by Salinity

Salinity is one of the major constraints in wheat growth, development, and production. To investigate the ameliorative effect of exogenous SA and/or SPM on the growth and productivity of two wheat cultivars (Shandawel 1 and Sids 14) grown under saline circumstances, we measured the changes of leaf area, shoot dry weight, root dry weight, grain number, and grain yield in the SA- and/or SPM-treated plants subjected to non-saline and saline conditions.

**Figure 1:**
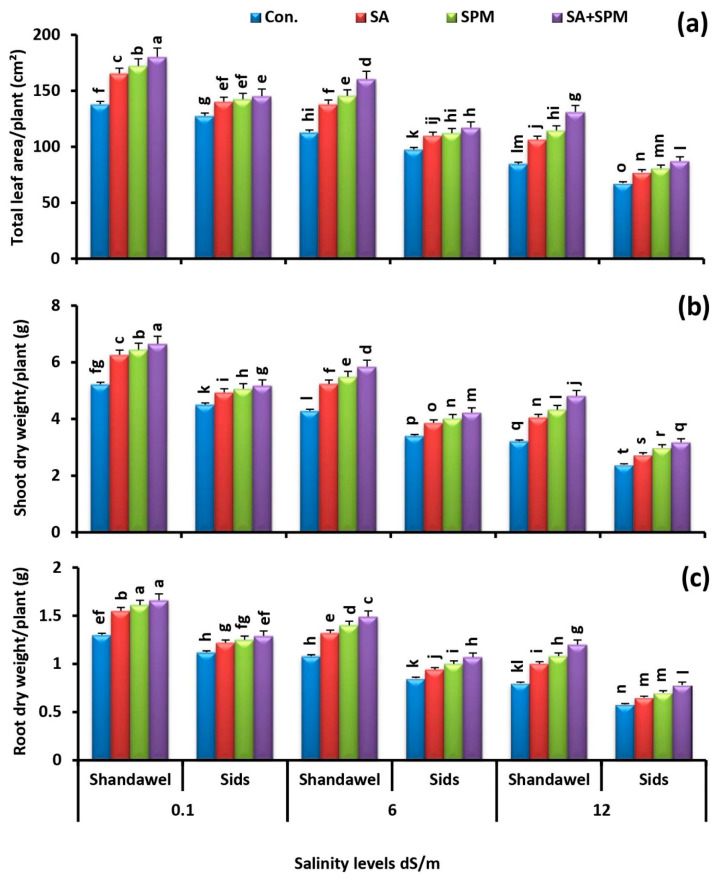
Influence of salicylic acid (SA; 100 mgL^−1^) and/or spermine (SPM; 30 mgL^−1^) on the (**a**) total leaf area, (**b**) shoot dry weight, and (**c**) root dry weight of two wheat cultivars (Shandawel 1 and Sids 14) grown under 0.1, 6, and 12 dS m^−1^ salinity levels. Data are mean of four replicates (n = 4). Different letters indicate significant differences at (*p* < 0.05) level, according to Duncan’s test.

Salt stress (6.0 and 12.0 dS m^–1^) has a major impact on wheat development. It suppressed wheat growth, resulting in a sharp reduction in the leaf area, shoot dry weight, and root dry weight, in both cultivars. These deleterious effects of saline conditions were prominent for cv. Sids 14 compared to cv. Shandawel 1. On the contrary, foliar applications of SA and/or SPM significantly mitigated this reduction compared with the salt-stressed plants that had not received any supplementations (Figure 1a–c). The combined treatment of SA and SPM yielded the best results, especially in Shandawel 1. It significantly (*p <* 0.05) elevated the leaf area (30.7%, 42.2%, and 55.0%), shoot dry weight (27.4%, 36.4%, and 49.8%), and root dry weight (27.7%, 38.0%, and 50.0%), in Shandawel 1 wheat plants subjected to 0.1, 6.0, and 12.0 dS m^–1^ salinity levels, respectively, compared with untreated plants.

**Figure 2:**
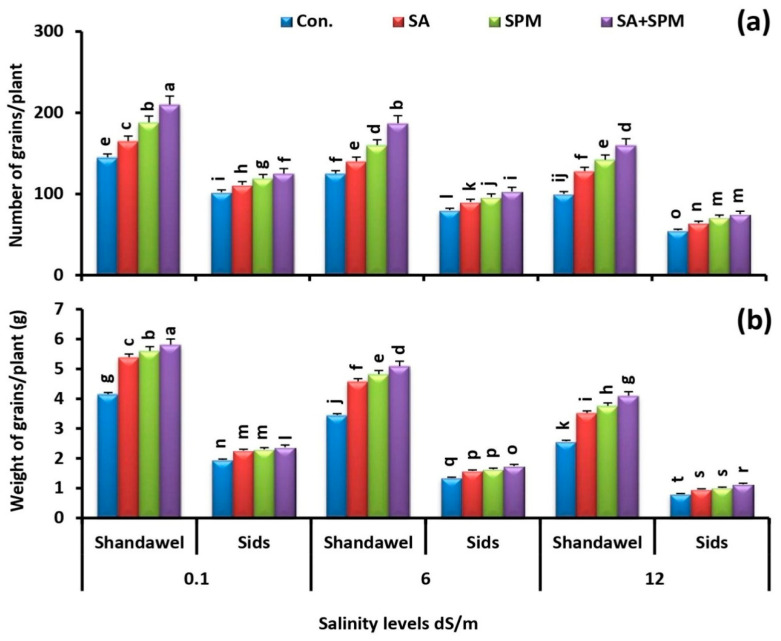
Influence of salicylic acid (SA; 100 mgL^−1^) and/or spermine (SPM; 30 mgL^−1^) on the (**a**) number of grains plant^−1^ and (**b**) weight of grains plant^−1^ of two wheat cultivars (Shandawel 1 and Sids 14) grown under 0.1, 6, and 12 dS m^−1^ salinity levels. Data are mean of four replicates (n = 4). Different letters indicate significant differences at (*p* < 0.05) level according to Duncan’s test.

In addition, as shown in Figure 2a,b, saline environments significantly reduced the productivity of cultivars Shandawel 1 and Sids 14 in terms of grain number and grain yield. These detrimental impacts of salt stress were prominent for cv. Sids 14 compared to cv. Shandawel 1. On the contrary, foliage applications of SA and/or SPM significantly alleviated the salt toxicity and attenuated the inhibitory impact of salt on these parameters. The best response was registered with SA and SPM combined treatment, especially in Shandawel 1. When compared to untreated plants, co-application of SA and SPM significantly (*p* < 0.05) increased the grain number by 44.8%, 49.6%, and 60.0% and grain yield by 40.0%, 47.5%, and 59.5% in Shandawel 1 wheat plants at 0.1, 6.0, and 12.0 dS m^−1^ salinity levels, respectively.

### 2.2. SA and/or SPM Treatments Enhance Photosynthetic Pigment Concentration in Salt-Stressed Wheat Plants

One of the most serious consequences of saline conditions is the decrease in the amount of photosynthetic pigments. To better understand whether SA and/or SPM foliage applications alleviate the damage of salt stress to the photosynthetic pigments, we measured the concentration of chlorophyll *a*, chlorophyll *b*, carotenoids, and total pigments along with the ratio of chlorophyll *a*/*b* in leaves of treated plants grown under non-saline and saline conditions.

**Figure 3:**
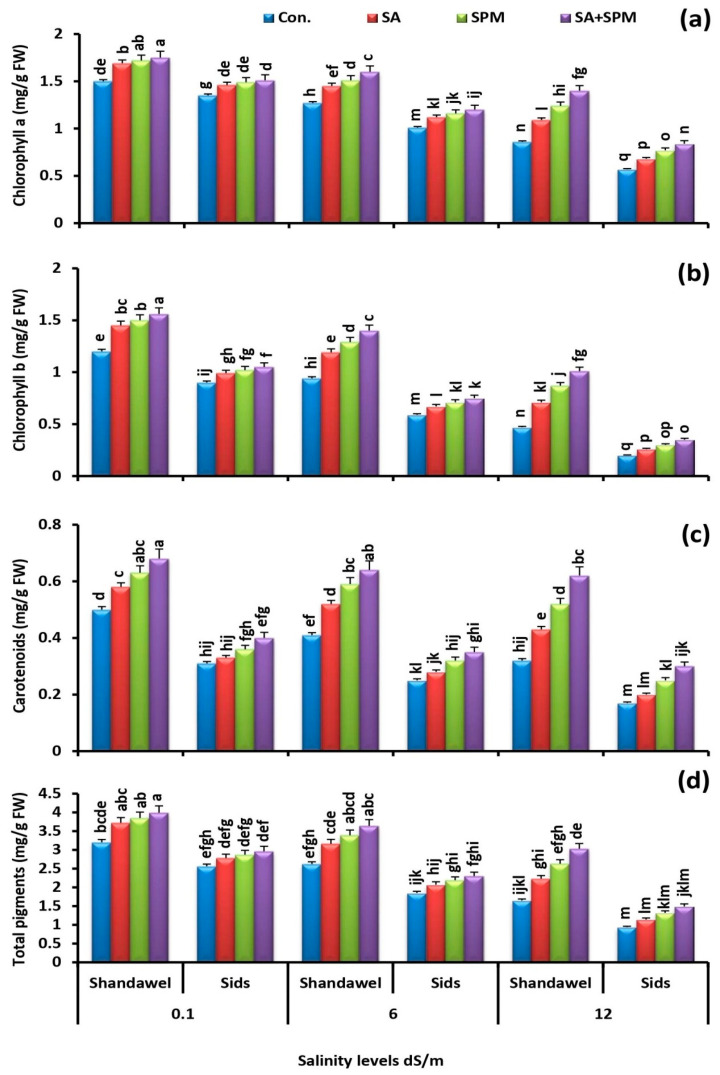
Influence of salicylic acid (SA; 100 mgL^−1^) and/or spermine (SPM; 30 mgL^−1^) on the concentration of (**a**) chlorophyll *a*, (**b**) chlorophyll *b*, (**c**) carotenoids, and (**d**) total pigments in leaves of two wheat cultivars (Shandawel 1 and Sids 14) grown under 0.1, 6, and 12 dS m^−1^ salinity levels. Data are mean of four replicates (n = 4). Different letters indicate significant differences at (*p* < 0.05) level according to Duncan’s test.

Soil salinization sharply decreased the concentration of chlorophyll *a*, chlorophyll *b*, carotenoids, and total pigments in the leaves of the two wheat cultivars (Shandawel 1 and Sids 14). The deleterious effect of salt stress was prominent for cv. Sids 14 compared to cv. Shandawel 1. However, exogenously-applied SA and/or SPM minimized the detrimental effect of saline environments and increased the photosynthetic pigment level in the absence and presence of salt stress (Figure 3a–d). Co-application of SA and SPM had the greatest ameliorative effect, especially in Shandawel 1. When compared to untreated plants, SA and SPM combined treatment significantly (*p* < 0.05) increased the concentration of chlorophyll *a* by 16.7%, 26.0% and 62.8%, chlorophyll *b* by 30.0%, 48.9% and 114.9%, carotenoids by 36.0%, 56.1%, and 93.8%, and total pigments by 24.7%, 38.9% and 83.6% in Shandawel 1 wheat plants at 0.1, 6.0 and 12.0 dS m^−1^ salinity levels, respectively.

**Figure 4:**
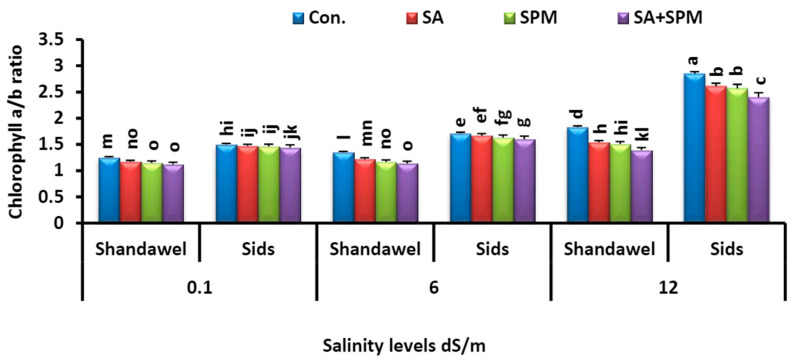
Influence of salicylic acid (SA; 100 mgL^−1^) and/or spermine (SPM; 30 mgL^−1^) on the chlorophyll *a*/*b* ratio in leaves of two wheat cultivars (Shandawel 1 and Sids 14) grown under 0.1, 6, and 12 dS m^−1^ salinity levels. Data are mean of four replicates (n = 4). Different letters indicate significant differences at (*p* < 0.05) level, according to Duncan’s test.

Furthermore, salt stress treatments (6.0 and 12.0 dS m^–1^) increased the chlorophyll *a*/*b* ratio in the leaves of both cultivars. On the contrary, comparing to the untreated stressed plants, the foliar treatments with SA and/or SPM significantly reduced the chlorophyll *a*/*b* ratio in the leaves of salt-stressed plants (Figure 4). The combined treatment of SA and SPM yielded the best results, especially in Shandawel 1. It significantly (*p <* 0.05) decreased the chlorophyll *a*/*b* ratio by 10.4%, 15.6%, and 24.0% in Shandawel 1 wheat plants subjected to 0.1, 6.0, and 12.0 dS m^–1^ salinity levels, respectively, compared with untreated plants.

### 2.3. Spraying of SA and/or SPM Maintain Ionic Balance in Salt-Stressed Wheat Plants

Ion toxicity is an important factor triggering severe damage to the growing plants under saline conditions. For exploring the influence of exogenous SA and/or SPM treatments on ions accumulation, we measured the accumulation profile of N, P, K^+^, Na^+^, Ca^2+^, Mg^2+^, Fe, Zn, and Cu in the grains of two wheat cultivars (Shandawel 1 and Sids 14) grown under non-saline and saline circumstances and sprayed with SA and/or SPM.

**Figure 5:**
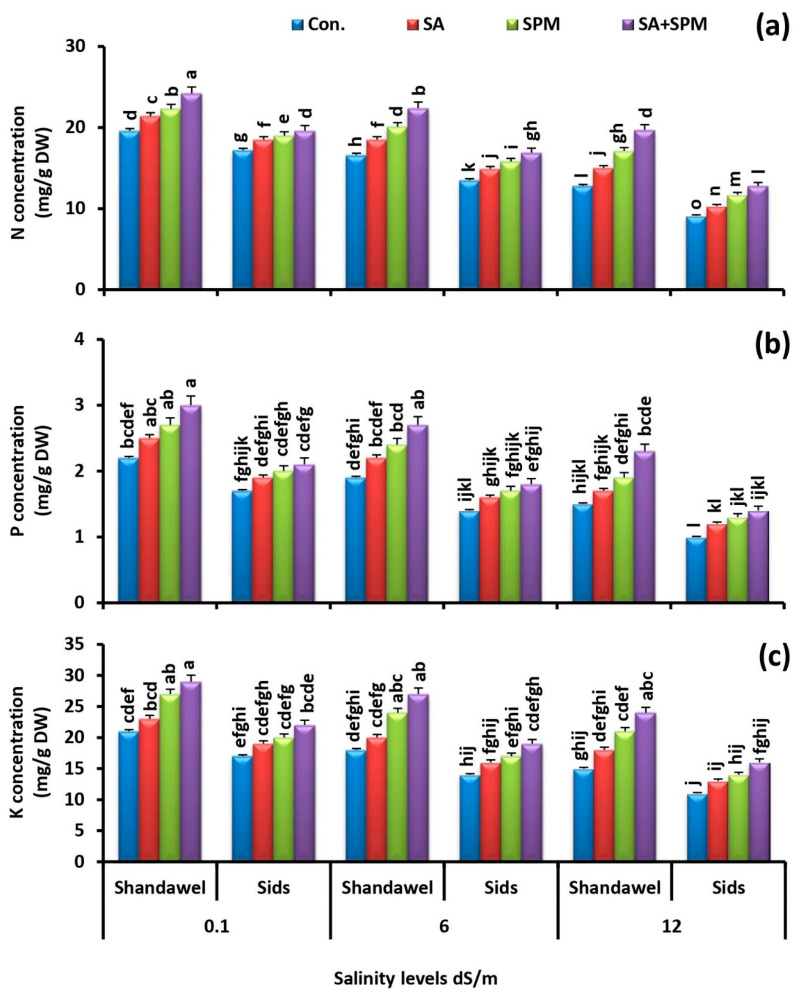
Influence of salicylic acid (SA; 100 mgL^−1^) and/or spermine (SPM; 30 mgL^−1^) on the concentration of (**a**) nitrogen, (**b**) phosphorus, and (**c**) potassium in grains of two wheat cultivars (Shandawel 1 and Sids 14) grown under 0.1, 6, and 12 dS m^−1^ salinity levels. Data are mean of four replicates (n = 4). Different letters indicate significant differences at (*p* < 0.05) level according to Duncan’s test.

Under saline conditions, the concentrations of N, P, and K^+^ were negatively affected. Moreover, this effect was more pronounced at high salinity level in both cultivars. Conversely, exogenous SA and/or SPM applications alleviated the deleterious injures of salt stress on mineral acquisition and increased the N, P, and K^+^ concentrations, especially in Shandawel 1 (Figure 5a–c). Co-application of SA and SPM yielded the best response and significantly (*p <* 0.05) increased the N concentration (23.5%, 34.9%, and 53.9%), P concentration (36.4%, 42.1%, and 53.3%), and K^+^ concentration (38.1%, 50.0%, and 60.0%) in grains of Shandawel 1 plants subjected to 0.1, 6.0, and 12.0 dS m^–1^ salinity levels, respectively, compared with untreated plants.

**Figure 6:**
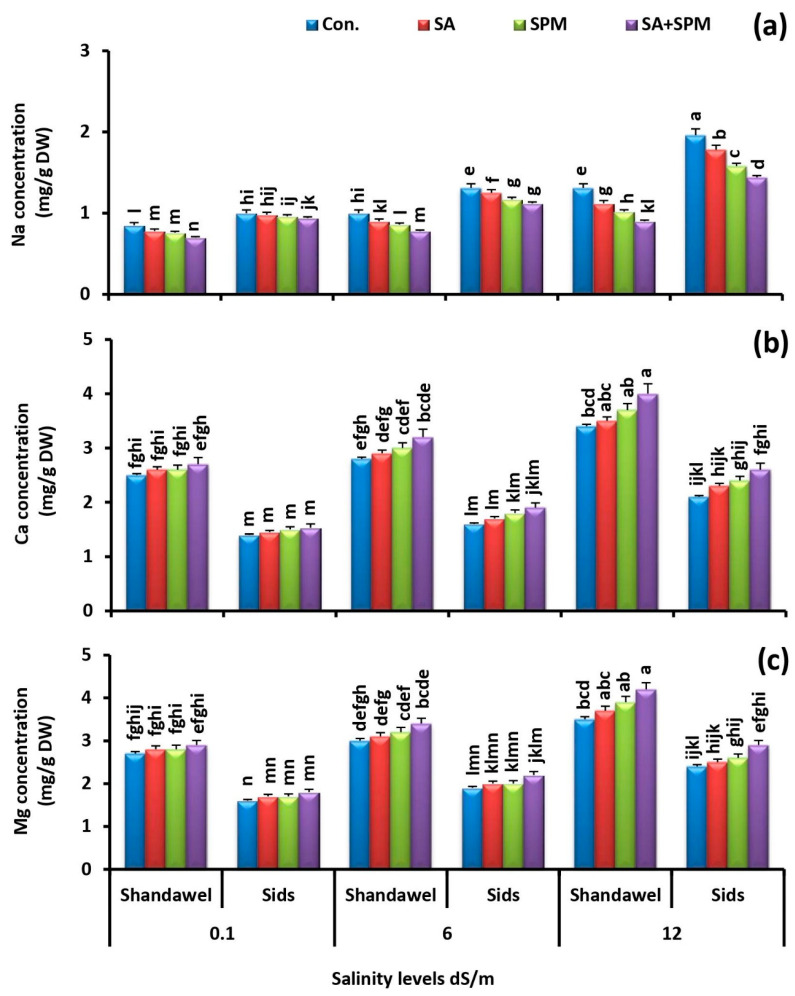
Influence of salicylic acid (SA; 100 mgL^−1^) and/or spermine (SPM; 30 mgL^−1^) on the concentration of (**a**) sodium, (**b**) calcium, and (**c**) magnesium in grains of two wheat cultivars (Shandawel 1 and Sids 14) grown under 0.1, 6, and 12 dS m^−1^ salinity levels. Data are mean of four replicates (n = 4). Different letters indicate significant differences at (*p* < 0.05) level, according to Duncan’s test.

Moreover, the Na^+^, Ca^2+^, and Mg^2+^ concentrations were increased by increasing the salt doses in the soil. However, the treatments with SA and/or SPM decreased the accumulation of Na^+^ while increasing the accumulation of Ca^2+^ and Mg^2+^ in the wheat grains (Figure 6a–c). The best response was registered with SA and SPM combined treatment, especially in Shandawel 1. At 6.0 and 12.0 dS m^–1^ salinity levels, the combination treatment significantly (*p <* 0.05) reduced Na^+^ values in grains of treated Shandawel 1 plants by 22.0% and 31.3%, respectively, compared to untreated plants.

**Figure 7:**
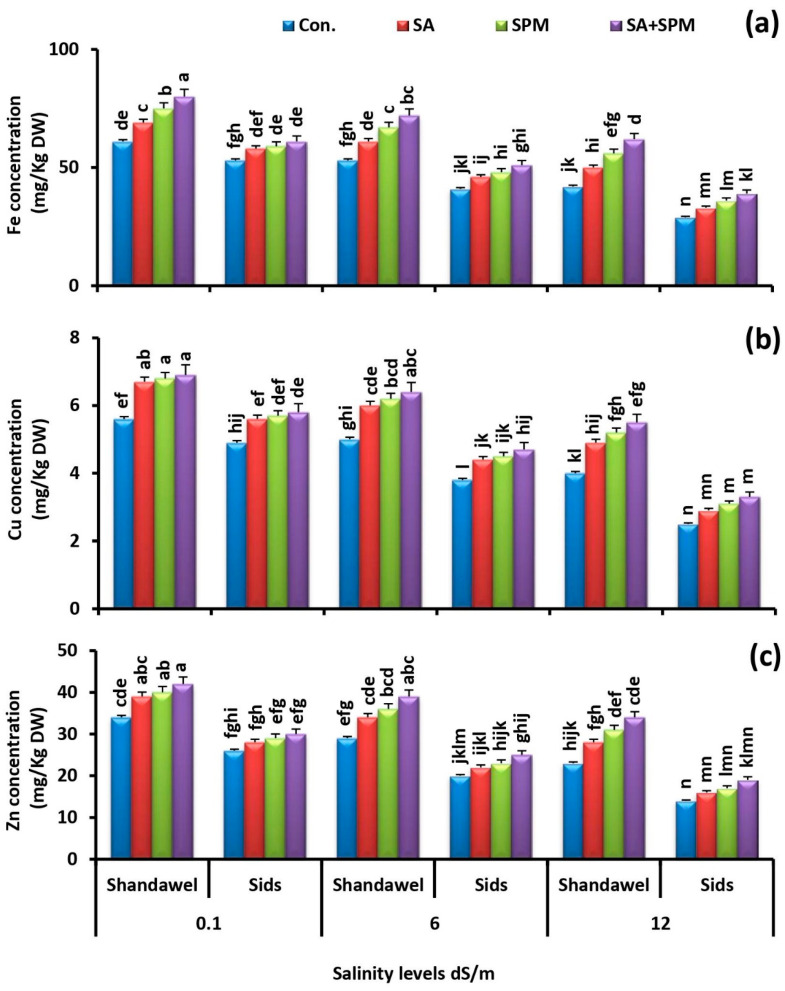
Influence of salicylic acid (SA; 100 mgL^−1^) and/or spermine (SPM; 30 mgL^−1^) on the concentration of (**a**) iron, (**b**) copper, and (**c**) zinc in grains of two wheat cultivars (Shandawel 1 and Sids 14) grown under 0.1, 6, and 12 dS m^−1^ salinity levels. Data are mean of four replicates (n = 4). Different letters indicate significant differences at (*p* < 0.05) level, according to Duncan’s test.

Additionally, under soil salinization, the concentrations of Fe, Zn, and Cu were sharply decreased with increasing the salinity level. Conversely, foliar SA and/or SPM treatments ameliorated the deleterious injures of saline conditions on mineral acquisition and increased the Fe, Zn, and Cu concentrations, especially in Shandawel 1 (Figure 7a–c). Co-application of SA and SPM yielded the best response and significantly (*p <* 0.05) increased the Fe concentration by 31.1%, 35.8%, and 47.6%; Zn concentration by 23.5%, 34.5%, and 47.8%; and Cu concentration by 23.2%, 28.0%, and 37.5%, in grains of Shandawel 1 plants grown under 0.1, 6.0, and 12.0 dS m^–1^ salinity levels, respectively, compared with untreated plants.

### 2.4. Exogenously Applied SA and/or SPM Preserve K^+^/Na^+^, Ca^2+^/Na^+^, and Mg^2+^/Na^+^ Ratios under Saline Conditions

**Figure 8:**
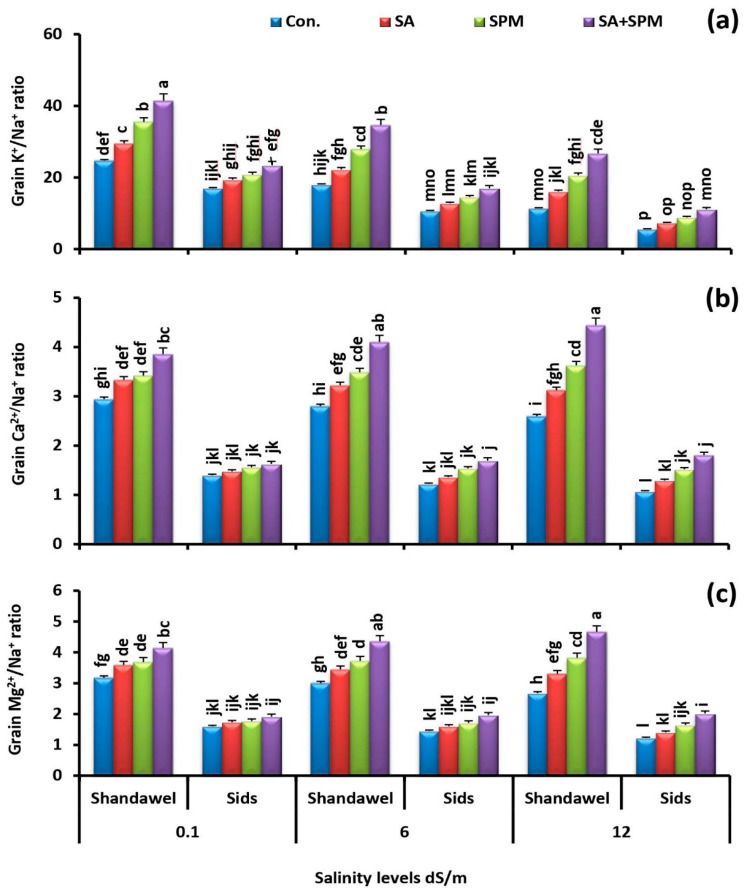
Influence of salicylic acid (SA; 100 mgL^−1^) and/or spermine (SPM; 30 mgL^−1^) on the ratio of (**a**) K^+^/Na^+^, (**b**) Ca^2+^/Na^+^, and (**c**) Mg^2+^/Na^+^ in grains of two wheat cultivars (Shandawel 1 and Sids 14) grown under 0.1, 6, and 12 dS m^−1^ salinity levels. Data are mean of four replicates (n = 4). Different letters indicate significant differences at (*p* < 0.05) level, according to Duncan’s test.

Salinity stress altered the ionic composition in wheat grains. To investigate whether the salt tolerance conferred by exogenous SA and/or SPM treatments, we quantified the K^+^/Na^+^, Ca^2+^/Na^+^, and Mg^2+^/Na^+^ ratios of two wheat cultivars (Shandawel 1 and Sids 14). Our findings showed that the K^+^/Na^+^, Ca^2+^/Na^+^, and Mg^2+^/Na^+^ ratios in the grains of salt-stressed plants were considerably lower than those in the grains of unstressed ones. On the contrary, SA and/or SPM applications sharply increased the ratios of K^+^/Na^+^, Ca^2+^/Na^+^, and Mg^2+^/Na^+^ in grains of stressed treated plants comparing to the stressed untreated ones (Figure 8a–c). Co-application of SA and SPM had the greatest ameliorative effect, especially in Shandawel 1. In comparison to values of untreated plants at salinity levels of 0.1, 6.0, and 12.0 dS m^–1^, coupling SA with SPM significantly (*p <* 0.05) increased the K^+^/Na^+^ ratio in grains of treated Shandawel 1 plants by 67.6%, 92.2%, and 132.2%; the Ca^2+^/Na^+^ ratio by 34.5%, 46.4%, and 69.2%; and the Mg^2+^/Na^+^ ratio by 28.1%, 46.7%, and 74.1%, respectively.

### 2.5. SA and/or SPM Treatments Improve Organic Solutes Accumulation under Salt Stress

Exposure to salinity induces the synthesis of organic osmolytes, which play a crucial role in counterbalancing Na^+^-induced reduction of water potential. Accumulation of osmoprotector solutes can protect plants from osmotic stress caused by salinity. To observe the change of compatible metabolites in the two wheat cultivars (Shandawel 1 and Sids 14) grown under different saline conditions and foliar sprayed with SA and/or SPM, we measured the concentration of total soluble sugars, total free amino acids, proline, and glycinebetaine in the leaves of treated plants grown under non-saline and saline environments.

**Figure 9:**
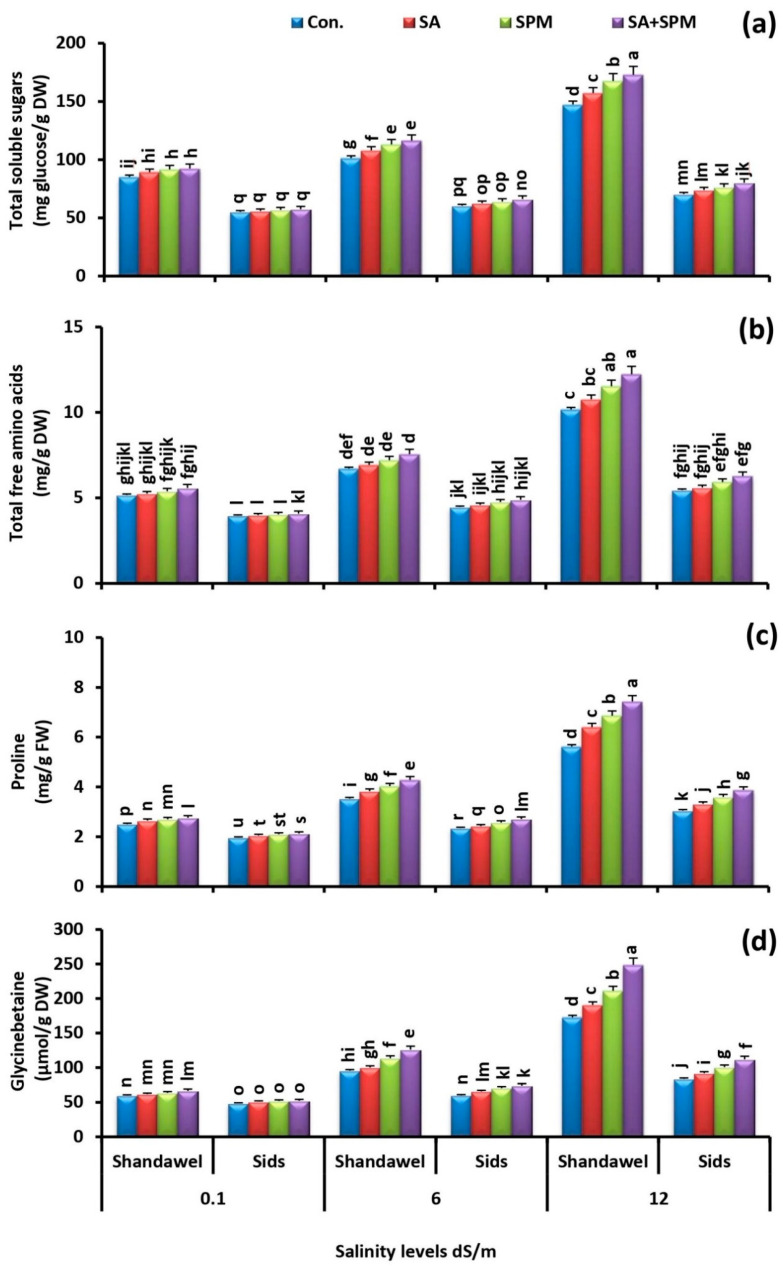
Influence of salicylic acid (SA; 100 mgL^−1^) and/or spermine (SPM; 30 mgL^−1^) on the concentration of (**a**) total soluble sugars, (**b**) total free amino acids, (**c**) proline, and (**d**) glycinebetaine in leaves of two wheat cultivars (Shandawel 1 and Sids 14) grown under 0.1, 6, and 12 dS m^−1^ salinity levels. Data are mean of four replicates (n = 4). Different letters indicate significant differences at (*p* < 0.05) level, according to Duncan’s test.

The accumulation of total soluble sugars, total free amino acids, proline, and glycinebetaine was increased dramatically in response to salinity treatments, as well as exogenous SA and/or SPM treatments in both cultivars (Figure 9a–d). The highest values were recorded in salt-stressed plants supplemented with a combination of SA and SPM, especially in Shandawel 1. Application of combined treatment under 6.0 and 12.0 dS m^−1^ salinity levels led to an increase in Shandawel 1 plants total soluble sugars concentration by 14.9% and 17.3%, total free amino acids concentration by 12.5% and 20.3%, proline concentration by 21.2% and 32.2%, and glycinebetaine concentration by 32.0% and 44.0%, respectively, relative to untreated plants.

### 2.6. Spraying of SA and/or SPM Enhance Grain Carbohydrate and Protein Content under Saline Conditions

Grains carbohydrate and protein content is important indicator of wheat grain quality, which is influenced by salinity stress, especially when wheat plants are exposed to saline environments at the grain filling stage. To investigate the ameliorative effect of exogenous SA and/or SPM on the grain quality of two wheat cultivars (Shandawel 1 and Sids 14) grown under saline circumstances, we measured the content of protein and carbohydrate in wheat grains of treated plants subjected to non-saline and saline conditions.

**Figure 10:**
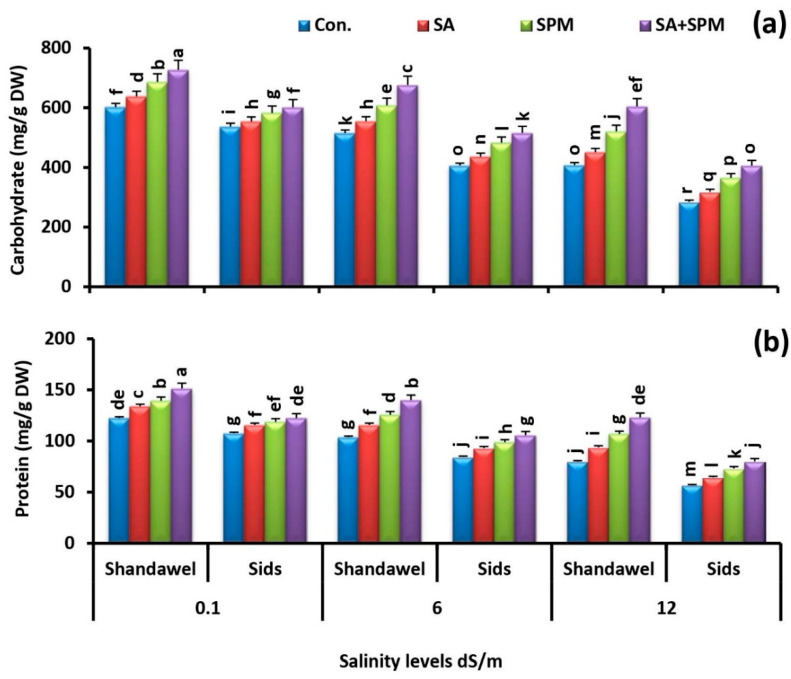
Influence of salicylic acid (SA; 100 mgL^−1^) and/or spermine (SPM; 30 mgL^−1^) on the content of (**a**) carbohydrate and (**b**) protein in grains of two wheat cultivars (Shandawel 1 and Sids 14) grown under 0.1, 6, and 12 dS m^−1^ salinity levels. Data are mean of four replicates (n = 4). Different letters indicate significant differences at (*p* < 0.05) level, according to Duncan’s test.

In view of the effect of salt treatments on grain carbohydrate and protein content, it was postulated that saline environments considerably reduced their values in both cultivars. The grains of salt-stressed plants had much lower amounts of carbohydrate and protein than those of unstressed ones. This deleterious effect of saline conditions was prominent for cv. Sids 14 compared to cv. Shandawel 1. On the contrary, under both saline and non-saline conditions, application of SA and/or SPM significantly (*p* < 0.05) enhanced their contents, especially in Shandawel 1 (Figure 10a,b). The combined treatment of SA and SPM had the greatest impact. When compared to untreated plants, co-application of SA and SPM significantly (*p* < 0.05) boosted carbohydrate content by 20.8%, 31.4%, and 48.2% and the protein content by 23.5%, 34.9%, and 53.9% in Shandawel 1 wheat plants at 0.1, 6.0, and 12.0 dS m^−1^ salinity levels, respectively.

## 3. Discussion

**Figure 11:**
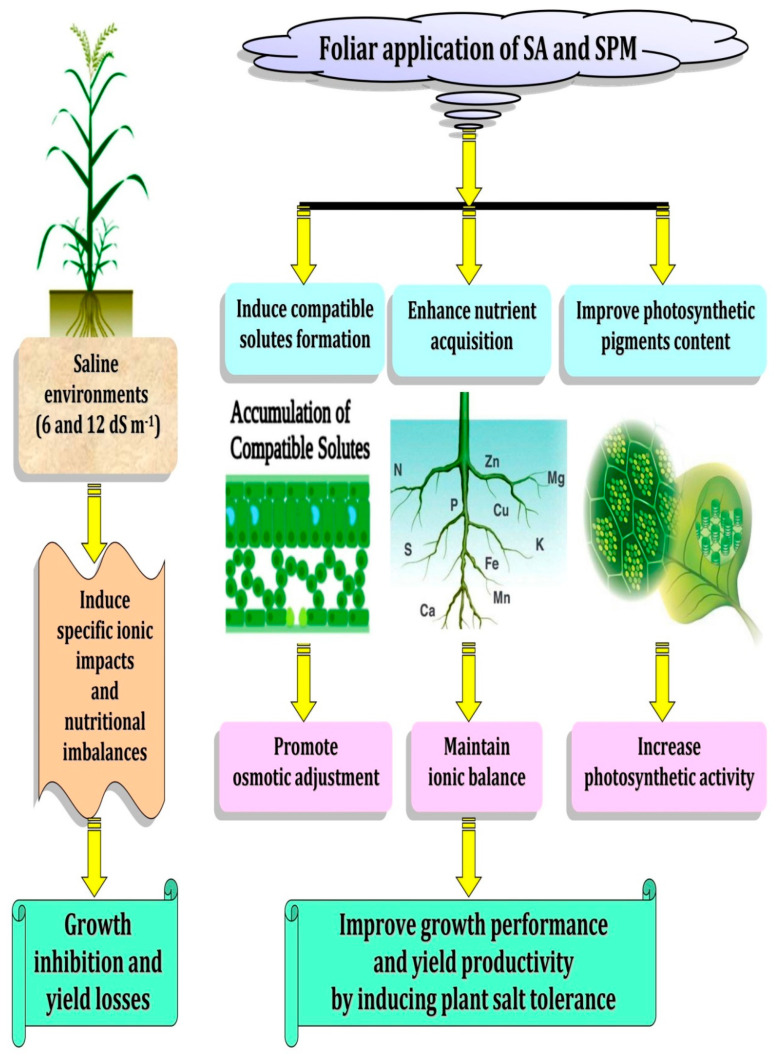
A model showing saline environments inhibit growth and productivity of wheat plants by inducing specific ionic effects and nutritional imbalances. Meanwhile, salicylic acid (SA) and spermine (SPM) reduce salt stress damage to the plant by improving photosynthetic pigment content, enhancing nutrient acquisition, and inducing osmolytes accumulation, thereby helping plants maintain ionic and osmotic balances.

Among all abiotic stresses, soil salinity is one of the major environmental constraints challenging crop production worldwide [1,4]. The excess amount of salts present in the agricultural land is considered as an immense threat to global food security [2,3]. Wheat constitutes pivotal position for ensuring food and nutritional security; however, rapidly rising soil and water salinity pose a serious threat to its production globally. Salinity stress negatively affects the growth and development of wheat resulting in restrained grain yield and quality [30,31]. Hence, improving wheat salt tolerance and exploiting arable areas of saline soils are becoming the most important scientific research and agricultural practices. Plant growth stimulators such as SA and SPM can play a key role in diverse plant growth and the development of physiological processes under different environmental stresses [13,14,23,26]. Some studies have looked into the effect of a single application of SA or SPM on plants exposed to salt stress. However, no one has looked into the impact of their combined treatment on wheat salt tolerance. To fill this gap, as a first investigation, we examined the effect of exogenously applied SA and/or SPM on photosynthetic pigment content, nutritional homeostasis, osmoprotectant synthesis, as well as grain carbohydrate and protein content in two wheat cultivars (Shandawel 1 and Sids 14) grown under non-saline and saline (6.0 and 12.0 dS m^–1^) conditions. Our findings clearly show that using SA and SPM together can reduce the negative effects of salt stress on wheat growth and production by enhancing photosynthetic pigment content, improving mineral acquisition, and promoting osmolytes accumulation (Figure 11). This research provides novel insights into the mechanisms of SA- and SPM-mediated amelioration of salt stress on wheat.

Growth and yield reduction can be used to determine the extent of salt-induced damage to the plants [4,32]. In the current study, data showed that salt stress resulted in a significant decline in the growth and yield of wheat plants in terms of total leaf area, dry weights of shoot and root, grain number, and grain weight, and this reduction increased with the increase in salinity levels, especially in cv. Sids 14 compared to cv. Shandawel 1 (Figure 1a–c and Figure 2a,b). This deleterious effect of saline environments on plant growth and development could be the result of (a) reducing the photosynthetic pigment concentration that can suppresses the photosynthetic efficiency, (b) inducing the nutritional imbalance that was closely associated with the reduction of N, P, K^+^, Fe, Zn, and Cu acquisition, along with the improvement of Na^+^ accumulation, (c) inducing the specific ion toxicity, as indicated by the disturbance in K^+^/Na^+^, Ca^2+^/Na^+^, and Mg^2+^/Na^+^ ratios, as well as (d) decreasing the synthesis and translocation of photosynthates (metabolites) from source to sink organs as indicated by the reduction in grain carbohydrate and protein content. Previous studies have shown that salinity affects plant growth through nutritional imbalances and ionic toxicity and causes membrane dysfunction and attenuation of metabolic activity [32,33]. By contrast, in agreement with previous reports [7,10,15,16,23,26,27], we observed that foliage applications of SA and/or SPM significantly ameliorated the negative impacts of soil salinization on wheat growth and production via upregulating of the photosynthetic pigment content, maintaining the optimal mineral nutrition, motivating the plant osmotic adjustment, and enhancing the grain carbohydrate and protein content. These findings clearly support the effectiveness of SA and/or SPM foliar treatments in attenuating the inhibitory effect of salt stress on plant development. In line with our findings, previous research by Miao [18] suggested that SA not only enhanced absorption range and capacity of water and nutrition by accelerating root growth, but also increased carbohydrate accumulation by promoting leaf photosynthetic ability, thus contributing to dry matter accumulation in salt-stressed plants. Moreover, the SPM application reduced the salt-induced growth inhibition by improving the chlorophyll content, compatible solutes accumulation, K^+^ content, and K^+^/Na^+^ balance [28].

Photosynthetic pigment concentration is an indicator of the photosynthetic machinery integrity, with positive correlations to photosynthetic activity [22,34,35]. In the present investigation, we showed that the concentration of photosynthetic pigment was sharply decreased as the salt concentration in soil increased (Figure 3a–d), implying that salt stress may cause pigment oxidation and degradation, and hence reduce pigment concentration [36]. Noteworthily, exogenously applied SA and/or SPM proved favorable by reducing the negative impacts of saline conditions and enhanced chlorophyll content in the absence, as well as the presence of salt stress. Previously, several scientists reported that foliage applications of SA and/or SPM improved chlorophyll levels in both unstressed and stressed plants by protecting thylakoid membranes and regulating chlorophyll biosynthesis and degradation pathways [13,26]. It has also been reported that SA and SPM may boost the activity of enzymes involved in the manufacture of chlorophyll or reduce the malfunction of the photosynthetic system, hence reducing chlorophyll degradation [15,27]. The results obtained in this trial also revealed that SA and/or SPM applied to salt-stressed plants prevented further salt damage and preserved the carotenoid concentrations. Carotenoids are important non-enzymatic lipid soluble antioxidants that play multiple functions in plant metabolism, including oxidative stress tolerance, as they protect the chloroplast form the harmful ROS [37]. Indeed, SA and SPM’s beneficial effects on maintaining carotenoid amount may be directly related to their capacity to control cellular signaling, activate redox-sensitive regulatory pathways, and regulate processes in carotenoid production [13,22]. It is important to note that the growth improvement caused by SA and/or SPM treatments may be the result of an increase in the amount of photosynthetic pigments. Indeed, these applications could function as regulators to avert degradation of chlorophyll and carotenoid pigments and protect the photosynthetic apparatus, thus enhancing photosynthetic efficiency. 

Another effective strategy to resist salt stress employed by plants is to keep ion homeostasis and relieve ionic toxicity. Excessive soil Na^+^ absorbed and transported to the aboveground organs disturbs the intracellular ionic homeostasis in plants, damages the photosynthetic membrane structure, promotes the chlorophyll degradation, and affects the cytosolic enzyme activity, resulting in the restraint of plant growth and productivity [7,28]. In the current study, we found that saline conditions induced alteration of ion homeostasis, as shown by higher Na^+^ accumulation (Figure 6a) and lower N, P, K^+^, Fe, Zn, and Cu acquisition (Figure 5a–c and Figure 7a–c). Moreover, this effect was more pronounced at high salinity level. Excessive accumulation of Na^+^ interferes with various physiological processes in plants, thus changing the ion balance in cells and triggering ion damage to the plant [38]. By contrast, in agreement with the previous reports [23,26,28,39,40], we observed that foliage applications of SA and/or SPM relieved the adverse effects caused by salt stress and significantly improved N, P, K^+^, Fe, Zn, and Cu acquisition in wheat grains, indicating their regulatory role in enhancing mineral nutrition uptake, accumulation, and translocation. Furthermore, the results obtained in this trial also demonstrated that exogenous SA and/or SPM applications alleviated the deleterious injures of salt stress and reduced the Na^+^ accumulation, implying that the ability of plants to reduce ions influx into the cytoplasm is of great importance to salinity tolerance. Strong evidence has demonstrated that SPM can directly block non-selective cation channels from the cytosolic side, restricting Na^+^ penetration into cell [41]. SPM was also reported to improve the intracellular ion homeostasis by enhancing the salt overly sensitive (SOS) pathway (*AsSOS1*, *AsSOS2*, *AsSOS3*) and upregulating the transcript levels of the high affinity K^+^ transporters (*HKTs*: *AsHKT1*, *AsHKT2*, *AsHKT4*, *AsHKT6*, and *AsHKT7*) [28]. Plant high affinity K^+^ transporters can unload Na^+^ from the xylem, thus improving plant salt tolerance [42]. A pervious study has also shown that SPM can act as an endogenous regulator of cell K^+^ transport [43]. Furthermore, SA’s beneficial effect on maintaining ionic homeostasis could be directly linked to SA ability to induce H^+^-ATPase activity [44]. SA was also reported to prevent salt-induced K^+^ leakage through depolarization-activated-outward-rectifying K channels [45]. Generally, retaining high levels of K^+^ and lowering accumulation of Na^+^ in the cytosol are essential for increasing the salinity tolerance in plants [46]. Bearing in mind that, maintaining ion homeostasis by SA and/or SPM treatments could be contributed to better salt tolerance. Generally, our study’s results reveal that SA and/or SPM increase the nutrient acquisition in wheat grains, highlighting a substantial protective role of SA and/or SPM in maintaining the grain nutritional value under salt stress.

Salt stress induces specific ion toxicity, which was measured by the buildup of Na^+^, K^+^, Ca^2+^, and Mg^2+^. Na^+^ is the foremost toxic ion that typically accumulates during salt stress conditions interfering with K^+^, Ca^2+^, and Mg^2+^ uptake and transport, which causes disturbance to K^+^/Na^+^, Ca^2+^/Na^+^, and Mg^2+^/Na^+^ ratios [7,20]. A plant’s capacity to tolerate salt is directly correlated with its ability to keep adequate K^+^/Na^+^, Ca^2+^/Na^+^, and Mg^2+^/Na^+^ ratios [7,9]. In the present study, we found that, with increasing salt doses, these ratios were negatively affected (Figure 8a–c), resulting in poor plant growth and productivity. Under conditions of salt stress, Na^+^ enters the cytosol and depolarizes the plasma membrane, which results in continuous outflow of K^+^ and raises the Na^+^/K^+^ ratio in the cytosol [1]. Conversely, our results revealed significant increases in K^+^/Na^+^, Ca^2+^/Na^+^, and Mg^2+^/Na^+^ ratios in plants subjected to salty soils and sprayed with SA and/or SPM. This is consistent with a previous report, which demonstrated that the treatment with SA reduced Na^+^ accumulation, increased K^+^/Na^+^ ratio, and mitigated the negative effects of salt stress on plant growth and development [20]. SA’s beneficial impact on maintaining ionic homeostasis may be directly related to its ability to increase root H^+^-pump activity, which stimulates the input of K^+^ and improves K^+^/Na^+^ balance [7]. Previous investigation also provides evidence that SPM improves K^+^/Na^+^ homeostasis in barely by blocking Na^+^ influx into root epidermal and cortical cells and restricting K^+^ loss from shoots [47]. Most probably, the ability of stress treated plants to mitigate salt damage and survive under saline environments is achieved by maintaining a high selectivity for K^+^, Ca^2+^, and Mg^2+^ ions despite an excess of Na^+^ ions. In sum, our findings suggest that preserving ionic balance may be the key action of SA and/or SAM in preventing salt toxicity. Furthermore, the growth–promoter effects of SA and SPM might be the result of their role in ion homeostasis.

To overcome the osmotic stress induced by salinity, plants synthesize and accumulate compatible solutes, such as total soluble sugars, total free amino acids, proline, and glycinebetaine [13,39]. These organic solutes can induce osmotic balance and act as antioxidants [48,49]. Proline possesses antioxidant properties that act as chaperones to protect the structure of macromolecules from destruction when the cell is dehydrated [49]. In fact, proline can act as a free radical scavenger, a cell redox balancer, an enzyme protectant, and a cytosolic pH buffer stabilizer for subcellular structures [50,51]. Furthermore, glycinebetaine has a potential role in cell osmotic adjustment, membrane stabilization, and the detoxification of toxic ions in plants exposed to salt stress [49]. Similarly, a higher accumulation of soluble sugar is also beneficial to the osmotic regulation [52]. In this study, a high level of salt stress induced a sharp increase in the total soluble sugars, total free amino acids, proline, and glycinebetaine accumulation, and this increase was more pronounced in the leaves of Shandawel 1 plants than that in the leaves of Sids 14. Furthermore, the treatments with SA and/or SPM further increased their accumulation in wheat leaves, especially in Shandawel 1 (Figure 9a–d). In fact, the osmoprotector solute accumulation under salt stress has been linked to the stress tolerance in many plant species. Moreover, their concentrations have been reported to be greater in salt-tolerant plants than in salt-sensitive ones [20]. Much evidence showed that exogenous application of SA or SPM increases the synthesis of compatible osmolytes in the plant cells [13,22,26,39,51]. A previous report provides evidence that SA induces glycinebetaine accumulation under salinity stress and increases Na^+^ flux from cytoplasm to vacuole [53]. In addition, an exogenous application of SA induces proline accumulation to alleviate the deleterious effects of salinity [20]. According to earlier studies, SA causes the building of glycinebetaine by inhibiting the generation of ethylene [53] and causes the production of proline by boosting the activity of its biosynthesis enzymes [54]. SPM has been also proved to be an important regulator of tolerance to salt stress by inducing the buildup of the compatible osmotic substances [28], which is fairly in agreement with our findings. Based on these determined parameters, it is worth underlining that the application of SA and SPM to wheat plants’ exposure to salt stress improves their ability to synthesize osmolytes that helps them to survive under this harsh condition.

Excessive soil Na^+^ absorbed and transported to the aboveground organs disturbs intracellular ionic homeostasis in plants and reduces photosynthetic rate, biomass accumulation, and source-sink activity, which hastens the reproductive organs’ senescence [55]. It has also been observed that the unavailability of sufficient photo-assimilates during the reproductive stage is the leading cause for losing yield potential and affecting the grain quality of wheat [56]. Protein and carbohydrate content is the most important indicator of wheat grain quality and, hence, it governs and determines the endue quality. In the present investigation, we showed that the contents of proteins and carbohydrates in wheat grains were significantly decreased as a result of soil salinization (Figure 10a,b). Previous investigation provides evidence that the decrease in protein content of grains was due to the accumulation of salts in the root zone, which deteriorated the grain quality [55]. Similarly, higher concentration of Na^+^ in the external environment interferes with the absorption of N, which leads to lower protein content in wheat grains [56]. Furthermore, salt stress interfered with photosynthesis and reduced P concentration in plant shoots, which interfered with grain development and affected the cereals’ grain carbohydrate content [57]. Perhaps this reaffirms our hypothesis that the nutritional imbalance could be the major factor behind the deteriorated grain quality of wheat under salt stress. Interestingly, the results obtained in this trial revealed that, under both non-saline and saline circumstances, the SA and/or SPM treatments significantly increased the grain carbohydrate and protein content. It could possibly be due to their positive impact on (i) the photosynthetic pigment concentration that can improve the photosynthetic efficiency and (ii) the nutritional balance that was closely associated with the improvement of N, P, K^+^, Fe, Zn, and Cu acquisition, along with the reduction of Na^+^ accumulation. It is praiseworthy that increasing grain carbohydrate and protein content with SA and/or SPM applications sustains yield of wheat plants and maintains grain nutritional quality under salt stress.

Different wheat cultivars have different defense responses to salt stress, so it is important to investigate the beneficial impact of using the alleviating stressor agents, such as SA and SPM on different wheat cultivars. It is worth noting that wheat cultivars’ responses to SA and/or SPM applications in saline environments may vary. In our study, the magnitude of salt stress mitigation by SA and/or SPM appears to be stronger for Shandawel 1 than Sids 14, as evidenced by a higher increase in photosynthetic pigment (chlorophyll *a*, chlorophyll *b*, and carotenoids) content, nutrient (N, P, K^+^, Fe, Zn, and Cu) acquisition, osmolytes (total soluble sugars, total free amino acids, proline, and glycinebetaine) accumulation, grain carbohydrate and protein content, and a decrease in Na^+^ accumulation. As a result, we can suggest that, compared to Sids 14, Shandawel 1 was more responsive to exogenous applications of SA and/or SPM under salinity, resulting in greater improvement in photosynthetic pigment amount, ionic balance, osmolytes adjustment, and grain quality. It is widely accepted that positive ionic and osmotic responses to salt stress are widely accepted as signs of tolerance.

## 4. Materials and Methods

### 4.1. Plant Material and Growth Conditions

Wheat (*Triticum aestivum* L. cvs. Shandawel 1 and Sids 14) grains were obtained from the Wheat Research Department, Agriculture Research Center, Ministry of Agriculture, Egypt. Shandawel 1 and Sids 14 cultivars were selected based on their high yield productivity, and we tried to increase their salt tolerance by using SA and SPM foliar applications. Grains were sown in the plastic pots with 30 cm diameter and 35 cm height. Pots were filled with 15 kg of clay loamy soil (sand 37%, silt 28%, and clay 35%). For each pot, twelve grains thinned to six after germination were planted. Ammonium nitrate (33.5% N), calcium superphosphate (15.5% P_2_O_5_), and potassium sulfate (48% K_2_O) were applied at rates of 2.0, 2.0, and 0.5 g pot^−1^, respectively. In addition, 2.0 g pot^−1^ ammonium nitrate was added 30 days after planting. The pots were placed in the greenhouse of the Department of Plant Physiology, Faculty of Agriculture, Cairo University, Egypt, under natural light and temperature conditions, with average day/night temperature conditions of 22/16 ±2 °C and average humidity of 65%. The experiment was performed twice, on 10 September of 2019 and 2020, with consistent results. Before sowing, pots were divided into three groups. The first one was assigned as a control (non-saline; 0.1 dS m^–1^), and the other two groups were assigned as two levels of salinity treatment (6.0 and 12.0 dS m^–1^ salinity level; which obtained by adding to the soil a mixture of NaCl, CaCl_2_, and MgSO_4_ at the molar ratio of 2:2:1, respectively). Soil chemical analysis was carried out following the procedures of [58] and presented in Table 1.

The wheat plants at 50 days old (vegetative stage) and 100 days old (grain filling stage) from each salinity level were foliar sprayed with 0.00 (distilled water; DW), 100 mgL^−1^ SA, 30 mgL^−1^ SPM, and 100 mgL^−1^ SA + 30 mgL^−1^ SPM. SA and SPM concentrations were chosen according to the results of a preliminary experiment. Tween-20 (0.05%) was added as a surfactant at the time of treatment.

The experimental layout was a completely randomized design with three factors: two wheat cultivars (Shandawel 1 and Sids 14), three levels of salinity [0.1 dS m^–1^ (control), 6.0 and 12.0 dS m^–1^], and four spraying treatments [0.00 (distilled water; DW), 100 mgL^−1^ SA, 30 mgL^−1^ SPM, and 100 mgL^−1^ SA + 30 mgL^−1^ SPM]. Each treatment had four replicates.

### 4.2. Plant Growth and Productivity Analysis

The 75-days old plants were sampled (after 25 days of SA and/or SPM first applications) to measure total leaf area, shoot dry weight, and root dry weight. Total leaf area was estimated using a portable leaf area meter (LI-COR 3000, Lambda Instruments Corporation, Lincoln, NE, USA). For dry weight determination, plants were dried at 70°C for 48 h until a constant weight was obtained. At maturity, the number of grains and grain yield were recorded. Each treatment included four replicates, and each replication consists of six plants gathered from the same pot.

### 4.3. Determination of Photosynthetic Pigments Concentration

The concentration of photosynthetic pigments was determined in the upper leaves of 75-day-old wheat plants. Data were collected from four replicates, each of which contained six plants gathered from the same pot. Photosynthetic pigments from fresh leaves were extracted in 80% (*v*/*v*) acetone, and the concentrations of chlorophyll *a*, chlorophyll *b*, and carotenoids were determined spectrophotometrically according to the method described by [59] using a UV-1750 spectrophotometer (Shimadzu, Kyoto, Japan).

### 4.4. Determination of N, P, K, Na, Ca, Mg, Fe, Zn, and Cu Concentrations

The concentration of mineral ions was determined in wheat grains. Data were collected from four replicates, and each replication consists of six plants gathered from the same pot. Dried grains (0.5 g) were ground and digested in a mixture of boiling perchloric acid and hydrogen peroxide for 8 h until a transparent solution is obtained. Nitrogen concentration was obtained with the modified micro-Kjeldahl method following [60]. Phosphorus concentration was performed by the vanadomolybdophosphoric method [61]. Potassium and sodium concentrations were analyzed by a flame photometer (ELE UK). Elemental analyses of calcium, magnesium, iron, zinc, and copper were determined with an atomic–absorption spectrophotometer (Unicam 989-AA Spectrometer-UK).

### 4.5. Determination of Compatible Solutes Accumulation

The concentration of organic solutes was determined in upper leaves of 75-day-old wheat plants. Data were collected from four replicates, each of which contained six plants gathered from the same pot. Total soluble sugars, total free amino acids, and glycinebetaine concentrations were determined in dried ground leaves by the anthrone reagent method [62], ninhydrin reagent method [63], and the method of [64], respectively. Proline was determined in fresh leaf samples according to [65].

### 4.6. Estimation of Grain Carbohydrate and Protein Content

Carbohydrate and protein contents were determined in wheat grains. Data were collected from four replicates, and each replication consists of six plants gathered from the same pot. Determination of the content of carbohydrates and proteins in dried ground wheat grains were carried out according to [66,67], respectively.

### 4.7. Statistical Data Analysis

A completely randomized design was used with four replicates per treatment. Combined analysis was made for the two growing seasons since the results of the two seasons followed a similar trend. Three-way analysis of variance (ANOVA) was conducted using SAS software, version 9.4 (SAS Institute Inc., Cary, NC, USA). Differences between the treatments were tested by the Duncan test at a level of significance (*p* < 0.05).

## 5. Conclusions

Current findings reveal the important role of SA and SPM in improving the salt tolerance of wheat plants via upregulating the photosynthetic pigment level, motivating the plant osmotic adjustment, promoting the plant nutrient acquisition, and enhancing the grain quality. The SA and/or SPM foliar applications ameliorated the deleterious effect of salt stress on wheat growth and development by reinforcing the photosynthetic pigment (chlorophyll *a*, chlorophyll *b*, and carotenoids) content, improving the nutrient (N, P, K^+^, Ca^2+^, Mg^2+^, Fe, Zn, and Cu) acquisition, maintaining the ionic (K^+^/Na^+^, Ca^2+^/Na^+^, and Mg^2+^/Na^+^) homeostatic, inducing the osmolytes (total soluble sugars, total free amino acids, proline, and glycinebetaine) accumulation, enhancing the grain carbohydrate and protein content, and preventing the Na^+^ accumulation. The combined treatment of SA and SPM yielded the best results. Hence, this study explained the mechanism involved in salt stress alleviation by SA and/or SPM, which would help the researchers understand the importance of SA and SPM in mitigating salt stress in agronomic crops. Finally, we exposed a new environmentally friendly approach for farmers to stimulate the growth and productivity of their agronomic and horticultural crops under harsh environmental conditions.

## Figures and Tables

**Table 1:** Chemical properties of the soil under different salinity levels.

Salinity Levels EC (dS m^−1^)	pH	HCO_3_^−^ + CO_3_^2−^ (mg kg^−1^)	Cl^−^ (mg kg^−1^)	SO_4_^2−^ (mg kg^−1^)	Ca^2+^ (mg kg^−1^)	Mg^2+^ (mg kg^−1^)	Na^+^ (mg kg^−1^)	K^+^ (mg kg^−1^)
0.1	7.2	213.5	324.0	430.7	92.2	41.4	3.7	31.4
6.0	7.5	263.6	1173.4	996.9	398.5	173.9	306.7	39.7
12.0	7.8	275.4	1987.8	1686.1	886.5	314.5	808.6	52.6

## Data Availability

The data presented in this study are available in the article.
